# Methyl Gallate Suppresses the Migration, Invasion, and Epithelial-Mesenchymal Transition of Hepatocellular Carcinoma Cells *via* the AMPK/NF-κB Signaling Pathway *in vitro* and *in vivo*

**DOI:** 10.3389/fphar.2022.894285

**Published:** 2022-06-13

**Authors:** Huaguo Liang, Zexin Chen, Ruihui Yang, Qingsong Huang, Hongmei Chen, Wanting Chen, Li Zou, Peng Wei, Shijie Wei, Yongxia Yang, Yongli Zhang

**Affiliations:** ^1^ School of Life Sciences and Biopharmaceutics, Guangdong Pharmaceutical University, Guangzhou, China; ^2^ Guangzhou Key Laboratory of Construction and Application of New Drug Screening Model Systems, Guangdong Pharmaceutical University, Guangzhou, China; ^3^ School of Medical Information Engineering, Guangdong Pharmaceutical University, Guangzhou, China; ^4^ School of Pharmacy, Guangdong Pharmaceutical University, Guangzhou, China

**Keywords:** methyl gallate, migration, invasion, AMPK, epithelial-mesenchymal transition, hepatocellular carcinoma

## Abstract

Methyl gallate (MG), a polyphenolic compound found in plants, is widely used in traditional Chinese medicine. MG is known to alleviate several cancer symptoms. However, most studies that have reported the antitumor effects of MG have done so at the cellular level, and the inhibitory effect and therapeutic mechanism of MG in hepatocellular carcinoma (HCC) have not been extensively explored *in vivo*. We aimed to understand the therapeutic mechanism of MG in HCC *in vitro* and *in vivo*. MTT and colony formation assays were used to determine the impact of MG on the proliferation of a human HCC cell line, BEL-7402; wound healing and transwell assays were used to quantify the migration and invasion of HCC cells. Western blotting was used to quantify the expression of the AMPK/NF-κB signaling pathway proteins. *In vivo* tumor growth was measured in a xenograft tumor nude mouse model treated with MG, and hematoxylin–eosin staining and immunohistochemistry (IHC) were used to visualize the histological changes in the tumor tissue. We found that MG showed anti-proliferative effects both *in vitro* and *in vivo*. MG downregulated the protein expression of AMPK, NF-κB, p-NF-κB, and vimentin and upregulated the expression of E-cadherin in a dose-dependent manner. Additionally, MG inhibited the migration and invasion of HCC cells by decreasing MMP9 and MMP2 expression and increasing TIMP-2 expression. These were consistent with the results of IHC *in vivo*. MG inhibited the proliferation, migration, and invasion of HCC cells. This effect potentially involves the regulation of the AMPK/NF-κB pathway, which in turn impacts epithelial-mesenchymal transition and MMP expression.

## Introduction

Primary liver cancer is the third leading cause of cancer-related deaths worldwide, of which hepatocellular carcinoma (HCC) accounts for 90% of deaths (Llovet et al., 2021; Sung et al., 2021). Although, hepatectomy and liver transplantation are used as standard treatment strategies for HCC, they are only suitable for early-stage patients without cirrhosis (Anwanwan et al., 2020). Metastasis in the late stage leads to poor prognosis and high mortality rates (Zhu et al., 2019; Isik et al., 2020). Thus, there is an urgent need to identify natural drug components with low toxicity and high safety to replace traditional anti-HCC chemotherapy drugs and study the molecular mechanisms underlying the occurrence, development, and metastasis of HCC.

Methyl gallate (MG) is a polyphenolic compound that is derived from a variety of traditional Chinese medicinal plants such as *Juglans mandshurica, Paeonia lactiflora*, and *Galla chinensis* (Wu et al., 2021). It exhibits a wide range of biological activities, including antitumor, antioxidant, anti-inflammatory and antimicrobial effects (Chaudhuri et al., 2015; Correa et al., 2020; Ahmed et al., 2021). Previous studies have reported that MG induces autophagy and apoptosis in HCC cells, and when autophagy is inhibited, the cytotoxic effect of MG on HCC is markedly enhanced (Huang et al., 2021). In addition, MG blocked regulatory T cell infiltration in tumors and reduced resistance against cisplatin in lymphoma cells (Kim et al., 2016). In glioma cells, MG impeded cell migration by reducing the phosphorylation of Akt and ERK1/2 (Lee et al., 2013). However, the effect of MG on the proliferation and metastasis of BEL-7402 cells has not been fully studied, and the underlying molecular mechanism remains unclear. The AMPK/NF-κB signaling pathway is known to be closely related to tumor migration, invasion, and angiogenesis (Carling, 2017; Zhang et al., 2019; Hsu et al., 2021). Chen et al. (Chen et al., 2011) reported that exposure to an AMPK inhibitor inhibited NF-κB activity and blocked the migration of glioma cells. Several comprehensive studies have concluded that activation of the AMPK pathway is potentially carcinogenic and upregulates tumor cell proliferation in HCC (Liu et al., 2020). In addition, Chhipa R. R. et al. (Chhipa et al., 2018) showed high levels of AMPK expression in human glioblastoma, and the inhibition of AMPK reduced the viability of glioblastoma stem cells and tumors. Thus, key molecular players in the AMPK/NF-κB pathway may be the potential regulators of HCC pathophysiology and need to be further explored.

Extracellular matrix (ECM) degradation is critical for tumor metastasis (Garde and Sherwood, 2021). Pharmacological inhibition or knockdown of AMPK decreased the expression of MMP2 and MMP9 and prevented epithelial-mesenchymal transition (EMT), thereby blocking the migration and invasion of lung cancer cells (Hwang et al., 2021). During EMT, the connection between polarized epithelial cells and the basement membrane is weakened, thus enhancing migration and invasion capacities of cancer cells and making them more likely to infiltrate into the surrounding tissues (Mittal, 2018). Metastasis to distant sites also occurs due to blood circulation. This suggests that the upstream regulators of ECM molecules, such as MMP2 and MMP9, and inhibition of EMT can potentially reduce AMPK expression in cancers. Moreover, the degradation of ECM by MMPs is inhibited by the TIMP family proteins, preventing tumor invasion (Winer et al., 2018). The NF-κB transcription factor regulates the expression of TIMP-2 and is involved in tumor metastasis (Wisniewski et al., 2010). Therefore, cellular function, migration, and invasion of cancer cells and EMT may be regulated by MMP9 and MMP2, and the expressions of upstream regulators such as AMPK, NF-κB, and TIMPs need to be further explored.

In this study, we validated the antitumor effect of MG against HCC both *in vitro* and *in vivo*. Our study is also the first to reveal that MG inhibits the proliferation, migration, invasion, and the EMT of HCC cells by regulating MMPs and the AMPK/NF-κB pathway.

## Materials and Methods

### Chemicals

Methyl gallate (purity, ≥98%; molecular weight, 184.15; formula, C_8_H_8_O_5_) was obtained from Sigma-Aldrich (St. Louis, MO, United States). Methyl gallate stock solution was prepared using dimethyl sulfoxide (DMSO) (Sigma-Aldrich, St. Louis, MO, United States) and stored at −20°C. The final concentration of DMSO in the cell culture medium was less than 0.1% in all treatments.

### Cell Culture

Human hepatocellular carcinoma cell lines BEL-7402 and human normal hepatocytes LO2 were received from Fu Heng BioLogy (Shanghai, China). BEL-7402 cells were cultured in RPMI1640 medium containing 10% fetal bovine serum (FBS) (Sangon Biotech Co.,Ltd., Shanghai, China), and 1% penicillin/streptomycin (P/S) at 37°C in a humidified atmosphere of 5% CO_2_. LO2 cells were cultured in DMEM medium, and the conditions were consistent with BEL-7402 cells.

### Cell Viability Assay

Cell viability was measured using MTT assay. BEL-7402 cells were seeded in 96-well plates at 8,000 cells per well and treated with different concentrations of MG (20, 40, 80, 160, 320, and 640 μM) and 5-Fluorouracil (5-FU, 50 μM). After the specified time, 10 μL MTT (5 mg/ml) was added, incubated for 4 h at 37°C. The supernatant was discarded, and 150 µL of DMSO was added to every well and rotated for 10 min to dissolve the purple formazan crystals formed. Absorbance was measured on an microplate reader (AMR-100, Allsheng, China) at 490 nm.

### Colony Formation Assay

Inoculate 1,000 cells per well into 6-well plates overnight. Different concentrations of MG (0, 40, 80, 160 μM) and 5-FU (50 μM) were added to the medium. The medium is replaced every 5 days. After 2 weeks of culture, The cells were fixed using 4% paraformaldehyde (PFA) and then stained with 0.5% crystal violet (Meilunbio, Dalian, China). Wash 6-hole plate several times with ddH_2_O, dry and photograph.

### Wound Healing Assay

BEL-7402 cells were seeded into 6-well plates at 5×10^5^ cells per well, were incubated until 80–90% confluency. Using a sterile 200 μL pipette tip, a scratch wound was created by scraping the cell monolayer. BEL-7402 cells were washed three times with PBS, and the broken cells were washed. Then the cells were cultured in serum-free medium containing different concentrations of MG, and the images were obtained under microscope at 0, 24 and 48 h. The wound healing area was analyzed by Image-Pro Plus 6.0 (Media Cybernetics, Inc., Bethesda, MD).

### Transwell Cell Invasion Assay

The invasion was measured by Matrigel invasion chamber (Corning, NY, United States) with 8 μm pore size membrane. The bottom of the superior ventricle was precoated with 1 mg/ml 60 μL Matrigel (Lu et al., 2016). BEL-7402 cells were seeded (4 × 10^5^ cells/ml 200 µL) into the upper transwell chambers with serum-free RPMI1640. The RPMI1640 medium containing 20% FBS was added as a chemical inducer in the lower room. After incubation for 24 and 48 h, the cells in the upper room were gently scraped with cotton swabs, and the cells invading the submembrane surface were fixed and stained. Take pictures in 6 randomly selected fields of view under microscope (Nikon Eclipse E100, Japan).

### Western Blotting Analysis

RIPA buffer (Beyotime, China) was used to lyse cells to obtain total protein, and the protein concentration was determined by BCA method. The same amount of samples were separated by sodium dodecyl sulfate-polyacrylamide gel electrophoresis, and the protein was transferred to the PVDF membrane (Millipore, Burlington, United States). After blocking with 5% skim milk powder (Beyotime, China) for 2 h at room temperature, the membrane and antibody were incubated overnight at 4°C. The primary antibodies were as follows: AMPK, NF-κB, p-NF-κB, MMP9, MMP2, TIMP-2, E-cadherin, Vimentin and *β*-actin (Beyotime, China). Then incubated with HRP-labeled Goat Anti-Rabbit IgG (H + L) (Beyotime, China) at room temperature for 2 h. Protein bands were detected by enhanced chemiluminescence (Tanon chemiluminescent gel imaging system, Tanon-5200Multi, China).

### Xenograft Tumor Model

All animal experiments were approved by the Ethics Committee of the Guangdong Pharmaceutical University. Four-week-old male BAL B/C nude mice were procured from Guangdong Medical Laboratory Animal Center. After 1 week of acclimatization, 0.2 ml of BEL-7402 cells (1 × 10^7^ cells/ml) were subcutaneously injected into the right axillary region of the mice, followed by drug administration when tumor diameter was >3 mm. For drug treatments, mice were randomly divided into six groups. The control group was administered saline intraperitoneally (ip), the positive group was administered 5-FU (15 mg/kg, ip, three times a week), three experimental groups were administered different concentrations of MG (40, 80, 160 mg/kg/d, intragastric; ig), and the combination group was administered 5-FU and MG (5-FU, 15 mg/kg, ip, three times a week; MG, 80 mg/kg/d, ig). Tumor volume and body weight were measured every 3 days. Tumor volume was calculated as follows: (width^2^ × length)/2. After 21 days of drug administration, the mice were euthanized, and the xenografts, liver, and spleen were dissected and weighed. The average tumor weight, tumor inhibition rate (%), and organ weight ratio were calculated. Tumor inhibition rate (%) calculated by tumor weight. The tumor inhibition rate was calculated as: (control group tumor average weight—experimental group tumor average weight)/control group tumor average weight × 100%. The organ index of the liver and spleen was determined as organ weight (g)/body weight (g).

### Hematoxylin–Eosin (HE) Staining Assay

The tumor tissue was fixed with paraformaldehyde for 48 h, embedded in pure paraffin, and cut into sections of 4∼5 μm. Tissue sections were attached to slides, dewaxed in xylene, rehydrated in ethanol, and stained with hematoxylin and Eosin. The morphology of HE-stained tumor tissue was observed under an optical microscope (Nikon Eclipse E100, Japan).

### Immunohistochemistry Assay

Tumor tissue sections were dewaxed with xylene, then hydrated, and antigen repair was performed using citric acid (PH6.0) antigen repair solution (Servicebio, Wuhan, China). After blocking endogenous peroxidase, incubated with primary antibody at 4°C overnight, and then the tissue sections were incubated with secondary antibody at room temperature for 50 min. Finally, after DAB staining and neutral gum sealing, microscopic examination and image acquisition were performed. Quantitative analysis of IHC data was detected by Image-Pro Plus 6.0 (Media Cybernetics, Inc., Bethesda, MD).

### Statistical Analysis

All experimental results were expressed as the mean ± standard deviation (SD) of triplicate samples. Statistical analysis was performed using IBM SPSS Statistics 26.0 (IBM, Armonk, United States) and GraphPad Prism 8.0 (GraphPad Software, La Jolla, CA, United States). One-way analysis of variance and Dunnett multiple comparison test were used to determine the differences between the average values of different groups. Significance was determined at values *p < 0.05, **p < 0.01, ***p < 0.001.

## Results

### MG Inhibits the Proliferation of BEL-7402 Cells

**FIGURE 1:**
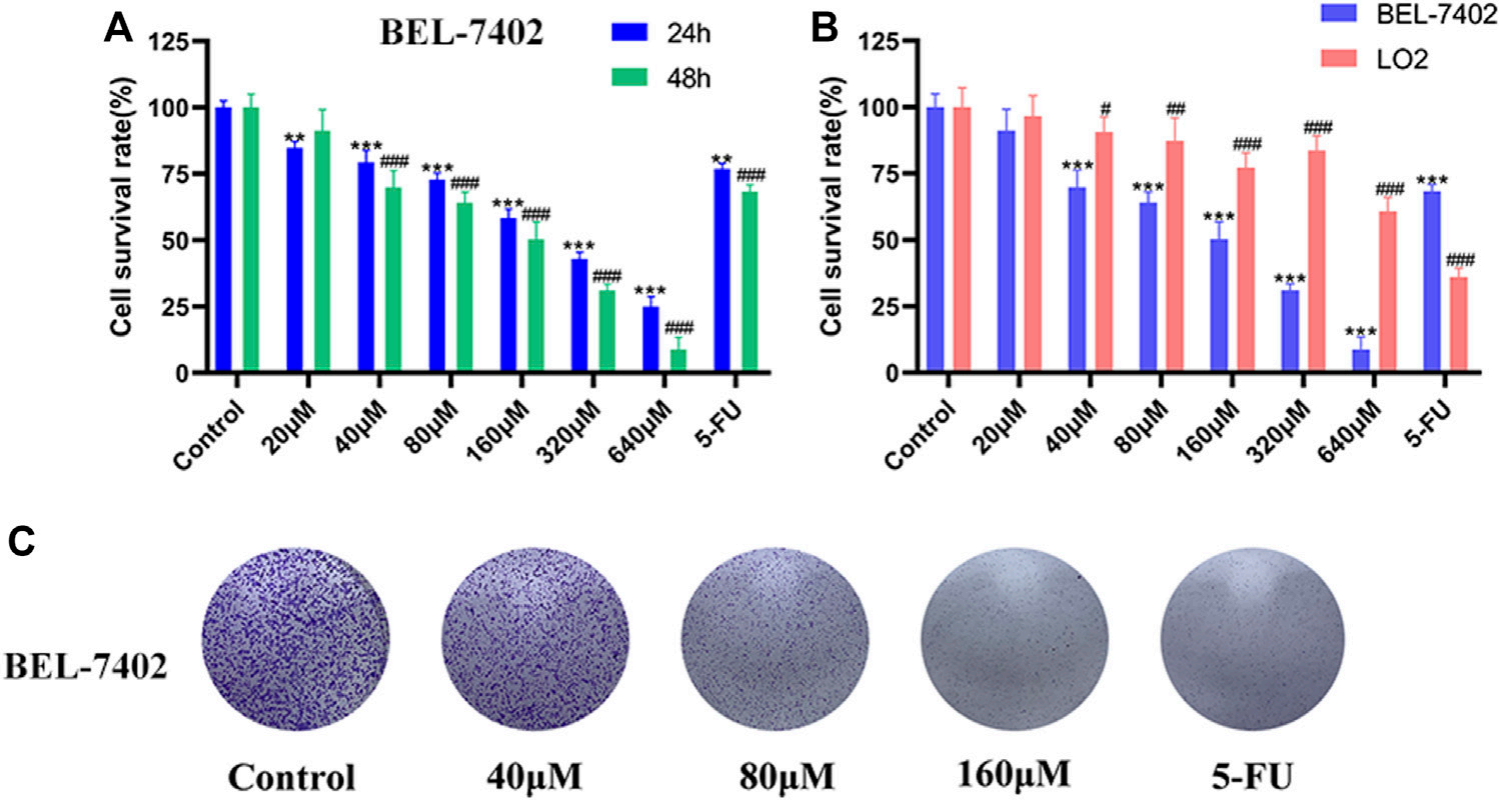
Effect of methyl gallate (MG) on cell viability. **(A)** BEL-7402 cells were treated with MG and 5-FU for 24 and 48 h **(B)** BEL-7402 and LO2 cells were treated with MG and 5-FU for 48 h. **(C)** The effect of MG on colony formation in BEL-7402 cells after 2 weeks of drug administration. Data were derived from at least three independent experiments. Data are presented as the mean ± standard deviation. *, ^#^ *p* < 0.05, **, ^##^ *p* < 0.01 and ***, ^###^ *p* < 0.001 vs. control.

The effect of MG (0–640 μM) on the proliferation of BEL-7402 and LO2 cells was quantified using the MTT assay, wherein 5-FU (50 μM) was used as the positive control. As shown in Figure 1A, MG inhibited BEL-7402 cell proliferation in a time- and concentration-dependent manner. Moreover, MG showed minimal toxicity in human normal hepatocyte LO2 cells (Figure 1B). The toxicity of MG in normal human liver cells, LO2 was less than 1/11th of that in the human liver cancer cell line, BEL-7402, and less than 1/7th of that of the positive drug 5-FU. These findings (Figures 1A,B) suggest that MG inhibits the proliferation of BEL-7402 cells *in vitro*, and it is far less toxic. Colony formation assay also showed that MG significantly inhibited the growth of BEL-7402 cells compared to that of the control cells (Figure 1C).

### MG Inhibits Migration and Invasion of BEL-7402 Cells

**FIGURE 2:**
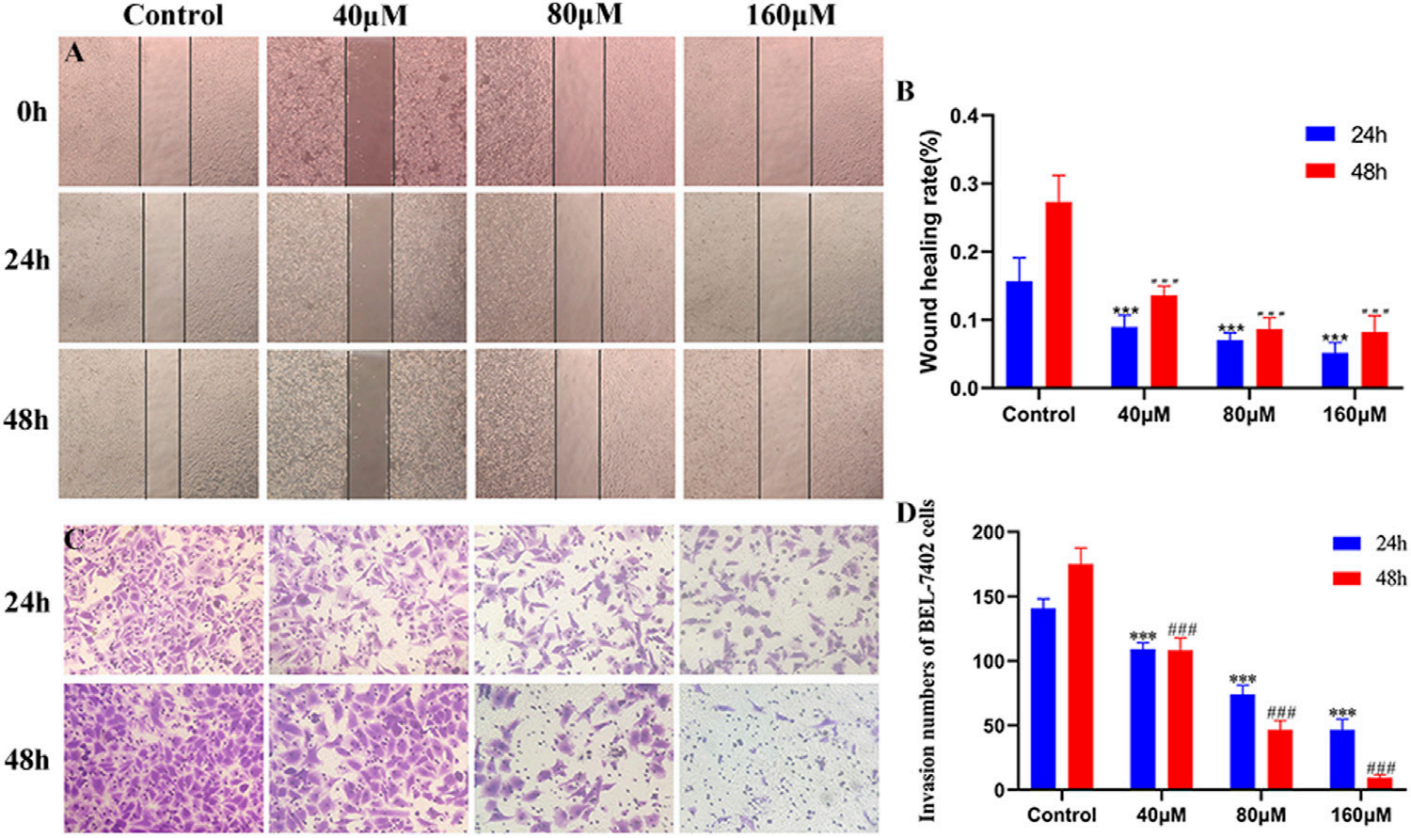
Inhibition of migration and invasion of BEL-7402 cells at different concentrations of methyl gallate (MG). **(A)** Representative images of wound healing in BEL-7402 cells treated with MG. **(B)** Quantitation of BEL-742 cells migration. **(C)** Transwell cell invasion in BEL-7402 cells treated with MG. **(D)** Quantitation of BEL-742 cell invasion. Data were derived from at least three independent experiments. Data are presented as the mean ± standard deviation. *, ^#^ *p* < 0.05, **, ^##^ *p* < 0.01 and ***, ^###^ *p* < 0.001 vs. control.

Cell migration and invasion are closely associated with tumor metastasis. As shown in Figures 2A,B, after 24 and 48 h of MG administration, scratch assay revealed that BEL-7402 cells in the control group healed gradually, and cell migration activity in the drug-treated groups reduced with an increase in MG concentration compared to that in control cells. Invasion ability was measured using the transwell cell invasion assay, wherein transwell pores were coated with Matrigel. The number of invaded cells in the MG-treated group was significantly lower than that in the control group. At 80 and 160 μM drug concentrations, the number of invaded cells after 24 h of treatment was significantly higher than that after 48 h of treatment (Figures 2C,D). These findings indicate that MG inhibits the migration and invasion of BEL-7402 cells in a time- and dose-dependent manner *in vitro*.

### Potential Mechanism of MG-Mediated Inhibition of Migration and Invasion of BEL-7402 Cells

**FIGURE 3:**
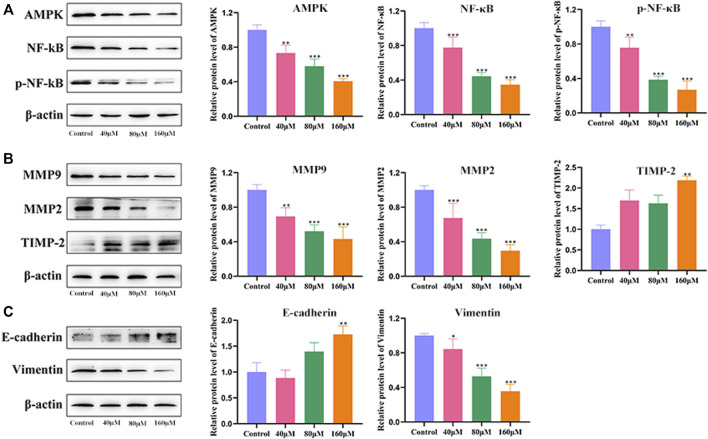
Effect of methyl gallate (MG) on migration, invasion, and related pathway proteins of BEL-7402 cells. **(A)** The influence of MG on protein expression of AMPK, NF-κB and p-NF-κB in BEL-7402 cells was quantified using western blotting. **(B)** The effect of MG on the expression of MMP9, MMP2 and TIMP-2 in BEL-7402 cells and the corresponding statistical analysis of protein levels. **(C)** Effect of MG on the expression of E-cadherin and Vimentin in BEL-7402 cells and statistical analysis of protein levels. Data from at least three independent experiments are presented as the mean ± standard deviation. *p < 0.05, **p < 0.01 and ***p < 0.001 vs. control.

To explore the underlying mechanism of MG-mediated inhibition of migration and invasion of HCC cells, BEL-7402 cells were treated with different concentrations of MG (40, 80, and 160 μM) for 48 h, and the protein expression levels of AMPK, NF-κB, p-NF-κB, MMP9, MMP2, TIMP-2, E-cadherin, and vimentin were quantified (Figure 3). MG treatment decreased the expression of AMPK, NF-κB, and p-NF-κB in a dose-dependent manner compared to that in the control group (Figure 3A). MMPs are essential for migration and invasion of cancer cells and degradation of the ECM (Scheau et al., 2019). Thus, we examined the inhibitory effect of MG on MMPs. Our results showed that MG significantly reduced the expression of MMP2 and MMP9 and increased the expression of TIMP-2, which is a negative regulator of cell matrix degradation, compared to that in the control group (Figure 3B). EMT is considered to be a critical event in cancer metastasis. EMT involves the downregulation of epithelial adhesion molecules, such as E-cadherin and cytokeratin, and upregulation of mesenchymal markers, such as vimentin and α-smooth muscle actin (*α*-SMA) (Brabletz et al., 2021). Our results showed that MG significantly increased E-cadherin and decreased vimentin protein expression at medium and high doses compared to that in the control group, significantly inhibiting EMT (Figure 3C).

### MG Inhibits the Growth of HCC Xenografts *in vivo*

**FIGURE 4:**
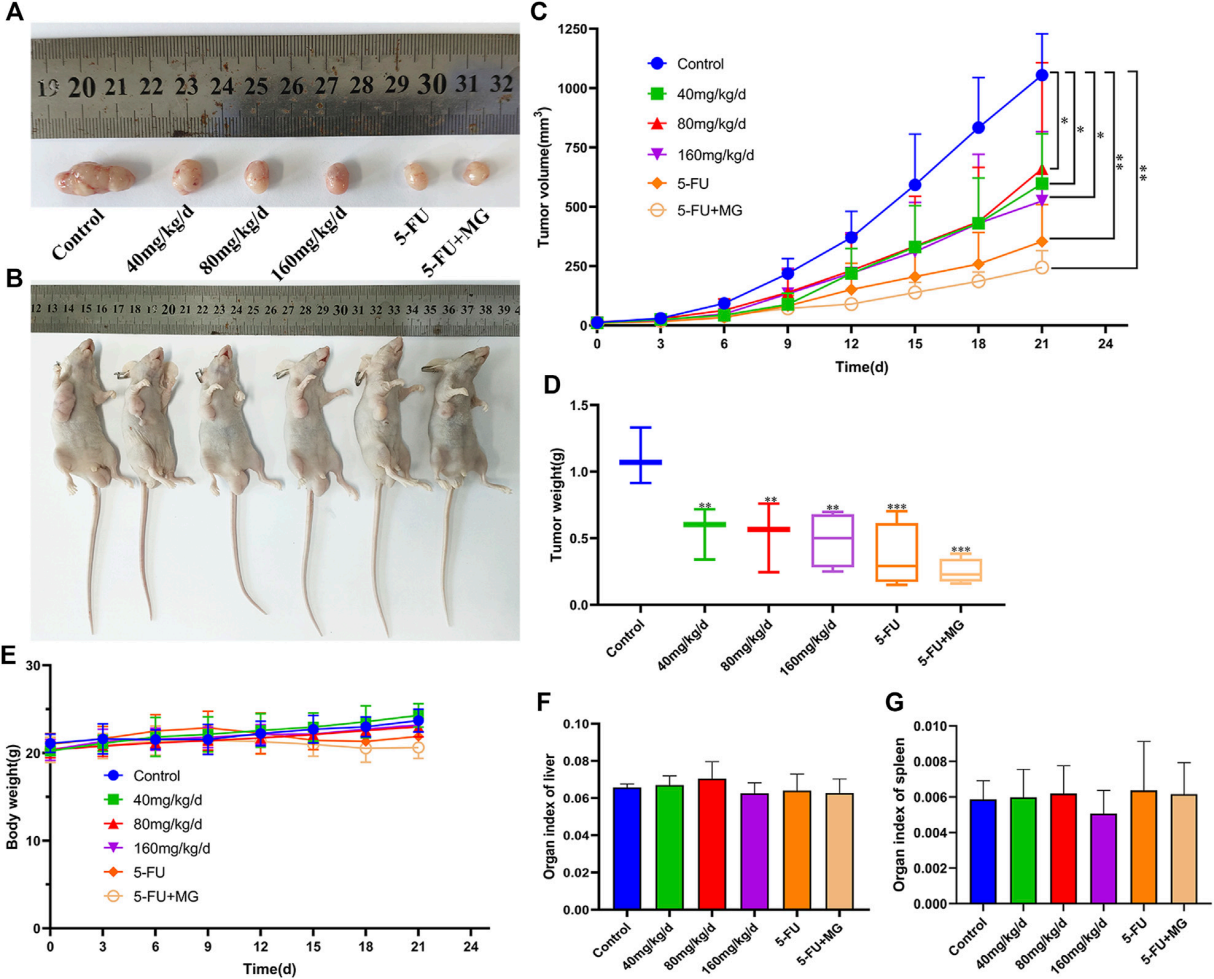
Methyl gallate (MG) inhibits tumor growth *in vivo* in BALB/C nude mice. **(A–B)** Tumor anatomy and physical appearance of nude mice treated with MG and 5-FU. **(C)** Analysis of tumor growth volume by MG and 5-FU. **(D)** Effect of MG and 5-FU on tumor weight. **(E)** Influence of MG and 5-FU on body weight of nude mice. **(F–G)** Effect of MG and 5-FU on liver and spleen indices of nude mice.

The safety and efficacy of MG *in vivo* were investigated in a xenograft model. The average tumor volumes and weights of the MG experimental group and the 5-FU positive group were significantly lower than those of the control group (Figures 4A–D). The tumor inhibition rates of 40, 80, 160 mg/kg/d of MG groups, 5-FU group, and combination group were found to be 49.94, 52.66, 55.92, 70.18, and 74.75%, respectively. Interestingly, the combination of MG and 5-FU had the strongest therapeutic effect, indicating that MG potentially promotes inhibition of tumor proliferation by 5-FU. Nonetheless, the mechanism requires further verification. MG and 5-FU had no effect on liver and spleen indices (Figures 4F,G). In the later stage of tumor induction and drug administration, it was observed that the body weight of nude mice in the MG 40, 80, and 160 mg/kg/d dose groups increased over time and was higher than that in the 5-FU and combined medication groups (Figure 4E). However, there was no significant physiological change in nude mice during the treatment, indicating that MG was well tolerated, relatively non-toxic, and safe for mice.

### Effect of MG on the Histology of Xenografts in Mice

**FIGURE 5:**
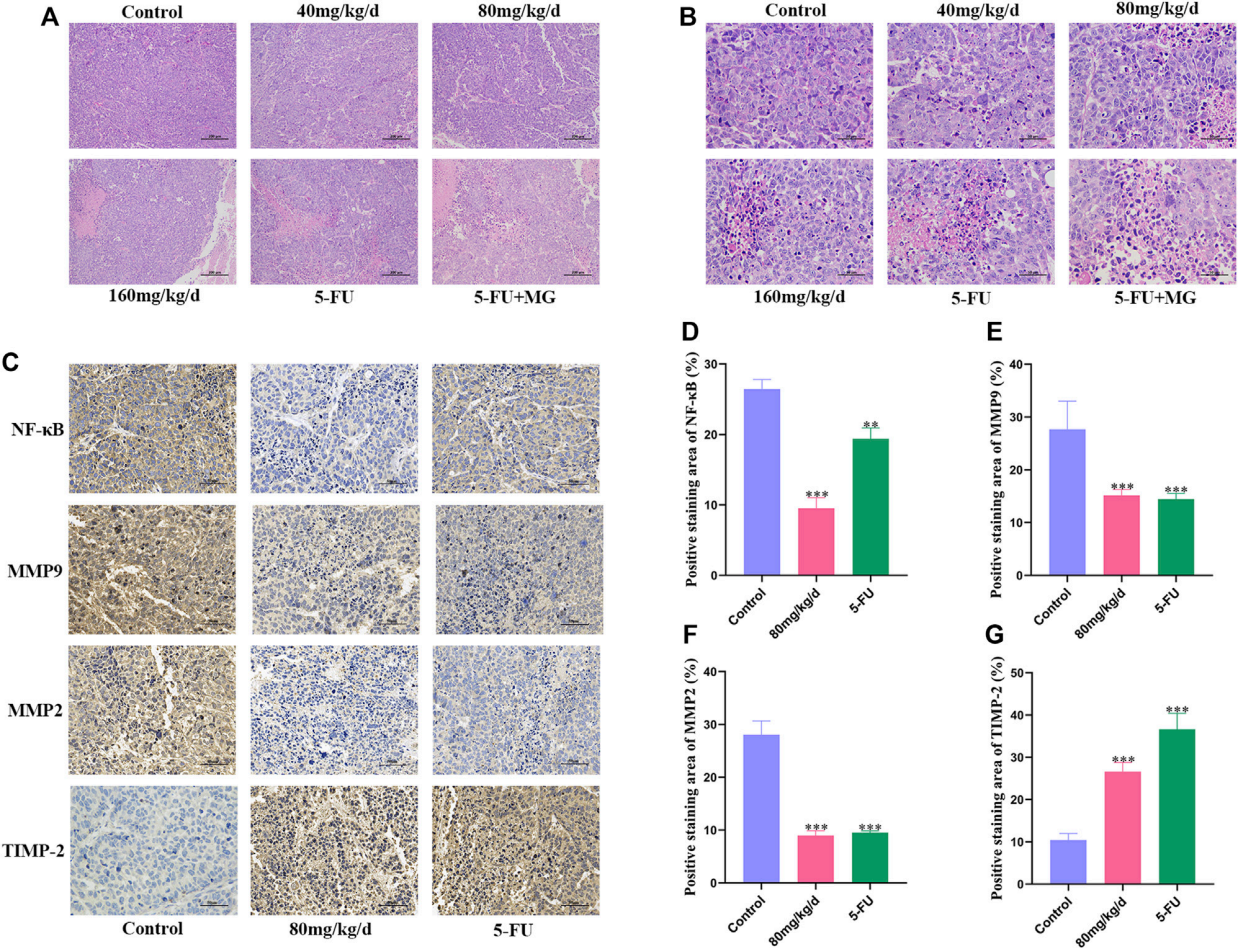
Effect of methyl gallate (MG) on histology of xenografts in nude mice. **(A–B)** Representative photograph of hematoxylin–eosin staining of xenografts, original magnification: **(A)** 10 ×; **(B)** 40 ×. **(C)** Immunohistochemical staining of NF-κB, MMP9, MMP2 and TIMP-2 in tumors of nude mice. **(D–G)** The positive staining results of NF-κB, MMP9, MMP2 and TIMP-2 were analyzed and quantified by Image J.

Pathological examination of xenografts from different groups was performed using HE staining. In the control group, we found large tumors with closely arranged cells, which were large in size with evident nuclear diversity and deep staining. After drug treatment, necrosis and apoptotic cells in tumor tissue gradually increased in size, cells were arranged loosely, and interstitial spaces increased. Furthermore, there was an increase in nuclear fragmentation, volume shrinkage, and chromatin condensation in the nucleus (Figures 5A,B). IHC was used to detect the expression of NF-κB, MMP9, MMP2, and TIMP-2, which are the four key targets for tumor migration and invasion. Compared with that in the control group, the expression of NF-κB, MMP9, and MMP2 in the 80 mg/kg/d MG group was significantly decreased, and the expression of TIMP-2 was markedly increased. The effects of 5-FU on these proteins were similar to those in the 80 mg/kg/d MG group (Figures 5C–G).

## Discussion

HCC is a common cause of cancer-related deaths worldwide (Sung et al., 2021). The occurrence and development of liver cancer is a complex process of several steps with multiple gene expression patterns that affect proliferation, differentiation, and metastasis (Narsinh et al., 2018; Bergers and Fendt, 2021). Particularly, the high frequency of malignancy and metastasis in the late stages of HCC results in the limited effectiveness of various treatment regimens. Several chemotherapy and molecular targeted drugs are used in the clinical treatment of liver cancer; however, the 5-year survival rate of patients treated with these drugs is extremely low (Reis et al., 2021). Therefore, there is an urgent need to identify new and more promising drugs or alternative preventive measures for the treatment of liver cancer (Ma et al., 2021). Natural plant drugs have multiple targets, less side effects, and good efficacy. MG is a polyphenolic compound found in natural Chinese medicinal plants that exhibits inhibitory effects on various tumors. This study aimed to examine the effect of MG on HCC and further explore its underlying molecular mechanisms. Our results show that MG significantly inhibited the growth of HCC both *in vitro* and *in vivo* and regulated EMT *via* the AMPK/NF-κB signaling pathway to inhibit the migration and invasion of BEL-7402 cells.

Recently, the role of AMPK in tumorigenesis and metastasis has begun to attract increasing attention. Studies have shown that AMPK plays an oncogenic role in liver cancer, glioblastoma, and esophageal squamous cell carcinoma (Monteverde et al., 2015). Inhibition of STAT1, which is regulated by AMPK, promotes the proliferation and metastasis of esophageal squamous cell carcinoma by triggering autophagy both *in vitro* and *in vivo* (Wu et al., 2020). In mouse breast cancer metastasis models, activated AMPK promotes cancer metastasis by aiding cancer cells to adapt to metabolic and oxidative stress (Cai et al., 2020). Thus, AMPK is thought to promote proliferation and metastasis of cancers. In addition, activated AMPK leads to the in the upregulation of FOXO1 and induces autophagy in cancer cells, promoting EMT (Xiao et al., 2020). Studies have also shown high levels of AMPK activation in lung adenocarcinoma patients with poor overall survival (Rackley et al., 2021). Interestingly, AMPK inhibitors inhibit NF-κB by inhibiting the phosphorylation of Ikkk*α*/*β* and p65Ser536 in glioma cells, thereby reducing cell migration (Chen et al., 2011). Previously, NF-κB was found to be constitutively activated in many cancers, and it promotes the survival, proliferation, and metastasis of tumor cells by increasing the expression of anti-apoptotic genes, cell cycle proteins, MMPs, cell adhesion genes, and angiogenesis-promoting genes (Li et al., 2015; Patel et al., 2018). Zhang Y et al. (Zhang et al., 2021) found that NF-κB signaling is activated by RUFY3 and induces EMT to promote cell growth, migration, and invasion in HCC. In contrast, when NF-κB translocation and transcriptional activity are inhibited, the expression of MMP2 and MMP9 is reduced and the expression of TIMP-2 is increased, further inhibiting the migration of cancer cells (Hsieh et al., 2017; Jin et al., 2021). Thus, AMPK is potentially one of the upstream molecules regulating NF-κB and EMT, and plays an essential role with regard to anticancer activity of MG. In this study, we found that MG downregulated AMPK expression in a dose-dependent manner, further inhibiting the activity of NF-κB and resulting in the inhibition of HCC progression.

**FIGURE 6:**
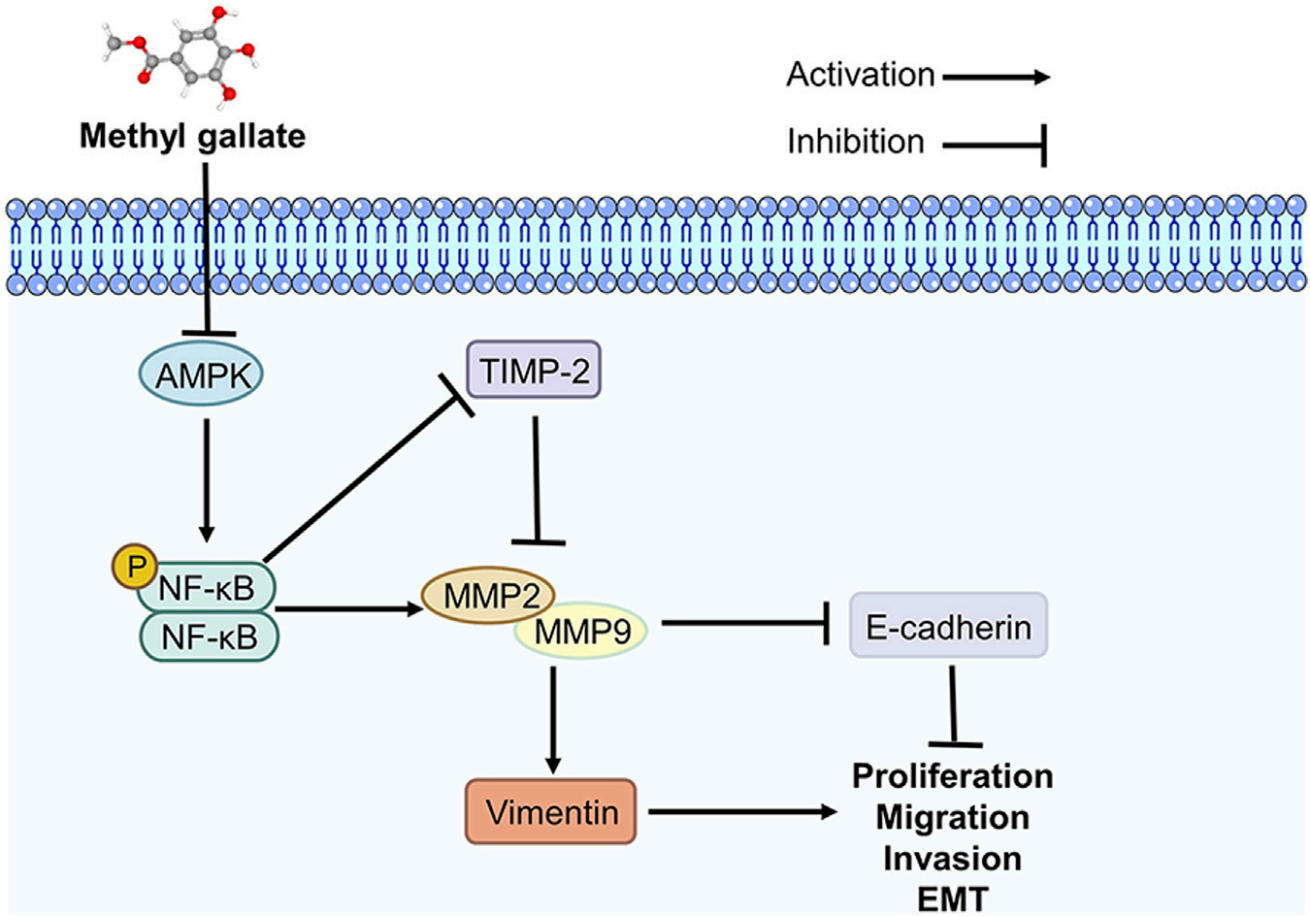
Methyl gallate inhibits migration, invasion and epithelial-mesenchymal transition of hepatocellular carcinoma through AMPK/NF-κB signaling pathway.

EMT plays a vital role in tumors and involves changes in the expression of multiple biomarkers. Vimentin and E-cadherin are important markers of EMT in cancer metastasis (Bhardwaj et al., 2020; Kaszak et al., 2020). Vimentin is primarily responsible for maintaining cytoskeletal integrity. Increased vimentin changes cell shape and increases cell motility, promoting the metastasis of cancer cells (Xuan et al., 2020). E-cadherin plays a crucial role in maintaining and establishing the epithelial integrity of the ECM. Loss of E-cadherin expression disrupts intercellular adhesion and connections, accelerating EMT (Corso et al., 2020). Therefore, these two targets were the focus of the present study. MMP2 and MMP9 are two of the main members of the MMP family that can downregulate the expression of E-cadherin and regulate the cleavage of E-cadherin to produce extracellular fragments, which are essential for tumor metastasis (Yang et al., 2018; Mondal et al., 2020). TIMP-2 regulates MMP-9 and MMP2 expression at the protein and mRNA levels and prevents further metastasis of tumor cells (Wang J. et al., 2019; Wang W. et al., 2019; Cabral-Pacheco et al., 2020; Kaczorowska et al., 2020). We found that MG suppresses the progression of HCC by regulating AMPK/NF-κB signaling pathway, and this has been summarized in Figure 6.

*In vitro* MTT assay showed that MG starkly impacted the viability of tumor cells; however, it was non-toxic to normal cells. Wound healing and transwell invasion assays showed that MG significantly inhibited the migration and invasion of HCC tumor cells, BEL-7402, in a dose-dependent manner. Western blot analysis further showed that MG treatment downregulated the expression of vimentin, MMP-2 and MMP-9, and upregulated the expression of E-cadherin and TIMP-2, indicating that MG significantly inhibited the EMT and ECM degradation, and prevented tumor metastasis. In addition, IHC assays *in vivo* also showed that MG inhibited the expression of NF-κB, MMP-2, and MMP-9 and upregulated TIMP-2 expression.

Results of western blotting and IHC were consistent with each other, indicating that our experimental results were reproducible and accurate. The molecular mechanism underlying the therapeutic effect of MG in cancers involves the downregulation of AMPK/NF-κB pathway proteins, thereby suppressing MMP expression and EMT. The transplantation tumor experiment in nude mice showed that the tumor inhibition rate induced by 40–160 mg/kg/d ig MG administration increased, in a dose-dependent manner, to more than 50%. In particular, the combination group of MG and 5-FU was safe and efficacious, showed a higher tumor inhibition rate than the positive 5-FU group, and had no significant effect on the physiological parameters of the nude mice, such as body weight. Our study provides clinical data for the potential use of MG and combinations of MG and other drugs in preventive and adjuvant therapies. However this study suffers from a few limitations. *In vitro* experiments involving the reverse regulation of AMPK/NF-κB pathway using inhibitors of downstream targets are currently in progress. Moreover, the therapeutic effect of MG on tumors in animals needs to be further evaluated and studied, including the IHC analysis of the other downstream targets of the AMPK/NF-κB pathway.

## Conclusion

In conclusion, we showed that the therapeutic effect of MG against HCC involves the downregulation of the AMPK/NF-κB pathway and MMPs, which in turn inhibit migration, invasion, and the EMT of tumor cells. Thus, our results suggest that MG, which is a naturally occurring bioactive molecule, holds great potential for use in treatment regimens against HCC and cancer.

## Data Availability

The raw data supporting the conclusion of this article will be made available by the authors, without undue reservation.
